# Imaging and Force Recognition of Single Molecular Behaviors Using Atomic Force Microscopy

**DOI:** 10.3390/s17010200

**Published:** 2017-01-22

**Authors:** Mi Li, Dan Dang, Lianqing Liu, Ning Xi, Yuechao Wang

**Affiliations:** 1 State Key Laboratory of Robotics, Shenyang Institute of Automation, Chinese Academy of Sciences, Shenyang 110016, China; limi@sia.cn (M.L.); ycwang@sia.cn (Y.W.); 2 School of Medical Device, Shenyang Pharmaceutical University, Shenyang 110016, China; dandangdan@sina.com; 3 Department of Industrial and Manufacturing Systems Engineering, The University of Hong Kong, Hong Kong, China

**Keywords:** atomic force microscopy, single-molecule, topography, mechanics, molecular recognition

## Abstract

The advent of atomic force microscopy (AFM) has provided a powerful tool for investigating the behaviors of single native biological molecules under physiological conditions. AFM can not only image the conformational changes of single biological molecules at work with sub-nanometer resolution, but also sense the specific interactions of individual molecular pair with piconewton force sensitivity. In the past decade, the performance of AFM has been greatly improved, which makes it widely used in biology to address diverse biomedical issues. Characterizing the behaviors of single molecules by AFM provides considerable novel insights into the underlying mechanisms guiding life activities, contributing much to cell and molecular biology. In this article, we review the recent developments of AFM studies in single-molecule assay. The related techniques involved in AFM single-molecule assay were firstly presented, and then the progress in several aspects (including molecular imaging, molecular mechanics, molecular recognition, and molecular activities on cell surface) was summarized. The challenges and future directions were also discussed.

## 1. Introduction

Cells are the structural and functional unit of living organisms. The cell is a hierarchically ordered system, which is composed of mutually interdependent species of molecules, molecular groupings, and supramolecular entities [1]. The diverse biological molecules residing on the cell surface or inside a cell play an important role in cellular physiological activities. For example, the cell surface molecules (e.g., receptors [2], sensors [3]) regulates many essential cellular processes, including cell adhesion, tissue development, cellular communication, inflammation, tumor metastasis, and microbial infection [4]. The malfunctions of membrane proteins often lead to the pathological changes in the cell (e.g., the B cell receptor in lymphoid malignancies [5]), and so far more than 50% of the approved drugs target human membrane proteins [6]. Inside the cell, the DNA methylation is involved in gene expression, protein function, and human diseases [7]. In cancer treatment, DNA-damaging chemotherapies have been the core for the past half-century [8]. Consequently, investigating the activities of biological molecules involved in cellular behaviors is of great significance in improving our understanding of life mysteries and related human diseases.

Traditional biochemical methods (e.g., X-ray crystallography [9], cryo-electron microscopy [10], and photoactivated localization microscopy [11]) require various pretreatments on the target molecules (e.g., purification, crystallization, and labeling), which inevitably cause damage to the natural structures of molecules. Besides, the obtained structures by X-ray crystallography and cryo-electron microscopy are essentially static, while the results acquired by photoactivated localization microscopy only reflect the behaviors of fluorescent spots rather than the target molecules. The advent of atomic force microscopy (AFM) provides an exciting tool for investigating the behaviors of single molecules in their native states, since AFM not only has sub-nanometer spatial resolution but also can work in aqueous conditions [12,13]. Conventional AFM takes minutes to record an AFM image, which is much greater than the time scale at which dynamic processes usually occur in biology [14]. In the past decade, due to the rapid development of high-speed AFM, the acquisition time of an AFM image was improved 1000-fold, and it is possible to take more than ten images per second, allowing us to clearly watch the real-time conformational changes of single biological molecules in action [15,16]. Besides topography imaging, via attaching antibodies (or ligands) to the surface of AFM tip, AFM can specifically recognize individual receptors on the cell surface by obtaining force curves on the cell surface, and this technique is termed single-molecule force spectroscopy (SMFS). SMFS can quantify the dynamic unbinding process of individual receptor-ligand pair, which is useful for reconstructing the folding energy landscapes of single molecules [17]. In recent years, multiparametric AFM based on peak force tapping (PFT) is also commercially available for simultaneously acquiring multiple physical parameters of samples, providing novel opportunities for investigating the correlation between the diverse properties of biological systems [18]. These developments greatly improve the performance and functions of AFM, widening the applications of AFM in life sciences and contributing much to the field of cell and molecular biology. There have been several in-depth review articles about applying AFM to detect the behaviors of single molecules, such as imaging molecular activities by high-speed AFM [19], characterizing cell membrane [20], studying membrane proteins and their interactions with ligands [21]. In this article, we will focus on the diverse methods and recent developments of applying AFM in single-molecule assay. Firstly the related techniques involved in AFM single-molecule assay are presented, and then the recent achievements in representative applications are summarized. Finally the challenges and future directions are discussed.

## 2. AFM Single-Molecule Techniques

**Figure 1:**
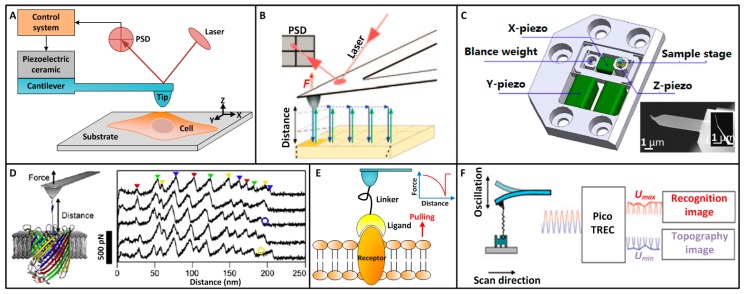
Typical AFM single-molecule techniques. (**A**) Principle of AFM. The tip raster scans the sample surface, during which the cantilever move vertically to maintain a constant interaction force between tip and sample. The force is detected by a laser reflected off the backside of the cantilever. (**B**) PFT multiparametric AFM imaging. The AFM tip approaches the withdraws from the sample in a pixel-for-pixel manner to record forces, *F*, over the tip-sample distance in force curves. The high precision of the approach allows detection of pixel sizes <1 nm^2^ with a positional accuracy of ~0.2 nm and forces at piconewton sensitivity. Reprinted with permission from [18]. Copyright 2013 Macmillan Publishers Limited. (**C**) Structure of a high-speed AFM scanner for narrow area (1 μm × 4 μm) imaging and scanning electron micrograph of a small cantilever for high-speed AFM. Reprinted with permission from [37]. Copyright 2014 Elsevier Ltd. (**D**) Mechanical unfolding of the membrane protein FhuA embedded in a lipid bilayer. A single FhuA is nonspecifically attached to the AFM tip. Increasing the distance of tip and membrane establishes a mechanical force that induces unfolding of FhuA. Force extension curves recording during unfolding a single FhuA show force peaks that measure the interactions established by unfolding intermediates of FhuA. Reprinted with permission from [49]. Copyright 2012 Elsevier Ltd. (**E**) Probing single receptors on cell surface with functionalized tip. A ligand is attached to the AFM tip and controlled to touch the receptor on the membrane. The receptor-ligand interaction is detected by pulling the receptor, during which force curve is recorded. The abrupt peak in the force curve corresponds to the receptor-ligand unbinding event. (**F**) TREC imaging. Special electronic circuit in the TREC box separates the maxima and minima of the oscillation amplitude during binding and generates the recognition and topographic image from them respectively. Reprinted with permission from [51]. Copyright 2016 American Chemical Society.

AFM uses a sharp tip mounted at the end of a cantilever to raster scan the surface of a sample to construct the topographical image of the sample, as shown in Figure 1A. The deflection of cantilever is detected by a four-quadrant position sensitivity detector (PSD) that senses a beam of laser reflected from the backside of the cantilever. According to Hooke’s law (*F* = *kx*, *k* is the spring constant of the AFM cantilever and *x* is the deflection of the cantilever), the interaction force between AFM tip and sample surface is acquired. During the contact mode scanning, according to the feedback control system, the piezoelectric ceramic driver controls the cantilever to move vertically to maintain a constant interaction force between AFM tip and sample surface by detecting the cantilever deflection. The forces involved in tip-sample interactions include van der Waals attractive force and electronic repulsive force. The contact mode scanning may cause damage to the sample due to the scratch. Tapping mode scanning eliminates the influence of lateral force on the sample by intermittently touching the sample. During tapping imaging, the amplitude of the vibrating cantilever is detected and the piezoelectric driver moves vertically to main a constant amplitude. The displacements of the AFM cantilever in vertical direction correspond to the topographical information of the sample surface. There are diverse types of AFM probes which are commercially available. Using adequate probes is important for single-molecule experiments, since it can influence the experimental results. The AFM probes used in certain references are summarized in Table A1 (see Appendix A). The unique advantage of AFM is that it can image the topography of the sample with high quality in liquids, making it very suited for observing biological samples, particularly the micro/nanostructures of living biological samples. For example, AFM can clearly visualize the individual microvilli [22], lipid rafts [23] and vesicles [24] on the surface of living cells. By attaching the native purple membrane onto mica, the detailed topography of individual bacteriorhodopsin molecules can be distinctly revealed by AFM imaging in buffer solution [25].

Recently, a new AFM imaging mode, which is called PFT [26,27], became commercially available for multiparametric imaging, as shown in Figure 1B. In the PFT mode, the vibrating tip indents the samples to record an array of force curves for each sampling points on the specimens. By real-time analyzing the different parts of the force curves, multiple parameters that reflect the physical properties of the samples are obtained. The Young’s modulus and adhesion force are obtained from the retract curve. The deformation is obtained from the approach curve. The energy dissipation is equal to the area between the approach curve and the retract curve. We know that conventional tapping mode reduces the influence of lateral force on the sample, but the vibration frequency of cantilever in conventional tapping mode is near the resonant frequency of the cantilever, which results in the relative large tapping force on the sample and thus may cause damage to fragile samples (such as living cells). In PFT, the vibration frequency of the cantilever is much less than its resonant frequency (e.g., the vibration frequency of cantilever in PFT in water is less than 2 kHz [28], while the resonant frequency of the cantilever in water is often larger than 10 kHz [29]). Hence, compared with normal tapping mode, PFT further decreases the tapping force between AFM tip and sample (the typical force ranges in conventional tapping mode are 1~2 nN [30], while the force ranges in PFT are 75~250 pN [22,31]), which is of active significance for probing living biological samples. For example, conventional tapping mode cannot reveal the individual microvilli on living kidney cells [32], while with the use of low force PFT (~100 pN) the single microvilli on living kidney cells have been clearly visualized for the first time [22]. By PFT imaging, the structural information of single native membrane proteins can be correlated to the mechanical properties [33], chemical properties [34], and electrostatic properties [35] of the proteins, providing novel insights into the behaviors of single molecules.

The life activities are dynamic in essence. There are many different types of molecules on the cell surface. These molecules do not work alone, but associate with each other in defined micro-and nanometer-scale regions of the cell membrane to fulfill various cell functions, such as cell adhesion, signaling, antigen presentation and cell-cell interactions [4]. Hence, visualizing the real-time molecular activities on the cell surface is of important significance for understanding life mysteries. The developments in high-speed AFM allow us to observe the behaviors of single molecules at work [15]. The basic configuration of high-speed AFM is consistent with that of conventional AFM. The differences between high-speed AFM and conventional AFM are that high-speed AFM achieves high-speed scanning by optimizing a series of parts, including cantilevers, cantilever deflection detection system, amplitude detector, high-speed scanner, dynamic PID control, drift compensator, and so on [36]. As shown in Figure 1C, the cantilevers of high-speed AFM are much more miniaturized (6–12 μm long) than conventional ones to achieve high resonant frequencies in water (400 kHz–1.2 MHz) and small spring constant (0.1–0.2 N/m) [37]; the scan range of the AFM head is limited to approximately 1 μm, 4 μm, and 1 μm in the X, Y, and Z directions to build a compact scanner and thus attain higher resonant frequencies and scan speeds [38]. With high-speed AFM, the dynamic activities of single myosin V molecules [12], single rotary motor proteins [39], single IgGs on bacterial surface [40], and single membrane proteins [41] were visualized, providing novel insights into molecular behaviors.

Besides isolated molecules on stiff substrates, in recent years applying high-speed AFM to directly imaging the live cells at single-molecule level has produced great achievements. In 2013, Colom et al. [42] investigated the activities of single proteins on lens cells using hybrid high-speed AFM/optical microscopy, clearly revealing the mobility of single proteins on cell membrane. In 2013, Suzuki et al. [43] revealed the dynamic events on single living cells by combining high-speed AFM with fluorescence microscopy, such as cell edge protrusion and membrane surface dynamics. In 2015, combining high-speed AFM with fluorescence microscopy, Yoshida et al. [44] successfully visualized the individual mitochondria and actin network structures on live cells and captured their dynamic changes. In 2015, by attaching an extremely long (~3 µm) and thin (~5 nm) tip by amorphous carbon to the cantilever, Shibata et al. [45] demonstrated the capability of high-speed AFM to observe the real-time changes of molecular activities on living cells, such as morphogenesis of filopodia, membrane ruffles, pit formation, and endocytosis. These studies [42,43,44,45] were obtained with narrow-area (<5 × 5 µm^2^) high-speed AFM. In 2013, Watanabe et al. [46] developed wide-area scanner with a maximum XY scan range of ~46 × 46 µm^2^ by magnifying the displacements of stack piezoelectric actuators using a leverage mechanism, revealing the dynamic bacteriolysis of single bacterial cells and the endocytosis occurring on HeLa cells.

Mechanically unfolding single membrane proteins by AFM provides a novel way to understand the dynamic processes of native proteins at the single-molecule level. Most proteins must fold into unique three-dimensional structures to perform their biological functions [47]. If protein misfolding occurs, and the misfolded proteins are not degraded, these proteins may form aggregated protofibrils that can cause diseases, such as neurodegenerative diseases, non-neuropathic systemic amyloidoses, and type II diabetes mellitus [6]. Mechanical forces are commonly employed for protein folding in a number of diverse proteins, and thus investigating the mechanical behavior of these proteins is of important significance for understanding protein folding [48]. AFM can mechanically unfold the single native proteins by obtaining force extension curves on the protein-contained lipid bilayer [49], as shown in Figure 1D. For mechanically unfolding membrane proteins, lipid bilayers containing reconstituted membrane proteins are adsorbed to flat substrates (e.g., freshly cleaved mica). AFM imaging is first performed to position the AFM tip over the membrane proteins of interest, and then the tip is pushed to the membrane protein until reaching a force of about 1 nN for 1 s, which allows the exposed polypeptide to adsorb to the AFM tip (with a probability about 15% [25]). Then the tip is retracted to induce protein unfolding while recording a force extension curve. The tip-protein binding is strong enough to allow the sequential unfolding of the membrane protein during retraction [50]. In the protein unfolding experiments, there is no control of which part of the protein attaches to AFM tip, and thus only those force extension curves reflecting the entire unfolding of the proteins (either the C- or N-terminal end of the protein attaches to the AFM tip) are used for analysis [25]. The unfolding of a membrane protein results in many sawtooth-like peaks in the force extension curve and each peak corresponds to a specific section of the folded protein. The recorded force extension curves are fitted by the worm-like chain (WLC) model to reveal the unfolding pathway of the protein [6,44]:

(1) $$F\left(x\right)=\frac{k_{B}T}{l_{p}}\left[\frac{1}{4}{\left(1-\frac{x}{L_{c}}\right)}^{-2}+\frac{x}{L_{c}}-\frac{1}{4}\right]$$

where *F*(*x*) is the force, *L_c_* is the contour length of the peptide, *l_p_* is the persistence length of the peptide (usually 0.4 nm for protein [6]), *x* is the extension of peptide, *k_B_* is Boltzmann’s constant, and *T* is the temperature. Each sawtooth-like peak is fitted by the WLC model. From the fitting, the number of amino acids is calculated from the contour length in WLC fitting (the contour length of an amino acid is 0.36 nm [25]), and then the unfolding pathway is obtained.

By using a functionalized tip, AFM can also recognize the individual membrane proteins on the cell surface and quantify the binding affinity of the proteins, as shown in Figure 1E. By performing approach-retract cycles on the cell surface with tip carrying ligands, force curves are recorded. If a ligand molecule binds to a receptor molecule during the contact between AFM tip and cell, the receptor-ligand pair is then pulled by AFM tip during the retract process. The receptor-ligand pair ruptures when the pulling force is larger than the binding energy, leading to a significant abrupt peak in the retract curve [52]. By controlling the density of ligands attached to the AFM tip to a low level, it can be confirmed that in each approach-retract cycle only one receptor-ligand pair forms [53], and thus the magnitude of the specific unbinding peak in the retract curve corresponds to unbinding force of a single receptor. Notably, for AFM force spectroscopy experiments, the spring constant of the cantilever should be calibrated to precisely quantify the molecular interactions. First, force curves are recorded on a stiff substrate (such as glass) to obtain the deflection sensitivity of the cantilever (nm/v). Then the spring constant of the cantilever is calibrated by thermal noise method [27]. The force resolution of AFM is determined by its spring constant. Hence, for single-molecule force measurements, best results are generally obtained with cantilevers exhibiting small spring constants (in the range of 0.01 to 0.1 N/m) and short lengths (<50 µm) [53]. By measuring the receptor-ligand unbinding force at different loading rates, the information about the dissociation dynamics of receptor-ligand interaction (e.g., the dissociation rate constant) and the prominent barriers traversed in the energy landscape along its force-drive dissociation pathways can be derived according to Bell model [54,55]:

(2) $$F=\frac{k_{B}T}{x}\ln\left(\frac{\gamma x}{k_{off}\left(0\right)k_{B}T}\right)$$

where *γ* is the loading rate, *k_off_*(0) is off-rate constant for dissociation in the absence of external force *F*, *x* is the position of the energy barrier that should be overcome during the dissociation, *k_B_* is the Boltzmann’s constant, *T* is temperature.

By obtaining arrays of force curves on the cell surface, the distributions of receptors can be mapped [56,57]. However, this method is time-wasting with a low efficiency. Researchers have developed a method to simultaneously obtain the topography and recognition information of the receptors on cell surface using functionalized tip, and this technique is called simultaneous topography and recognition (TREC) imaging [51], as shown in Figure 1F. When a ligand on the AFM tip binds to a receptor on the cell surface, the vibrating cantilever cannot return to its original position due to the pulling between AFM tip and the receptor-ligand pair, which causes the changes of the upper half of the cantilever’s oscillation signal but the lower half of the oscillation signal is not influenced. Hence, by separating the oscillation signal into the upper part (*U_max_*) and the lower part (*U_min_*) with a special electronic circuit, the recognition image and topography image can be simultaneously obtained.

## 3. Molecular Imaging

**Figure 2:**
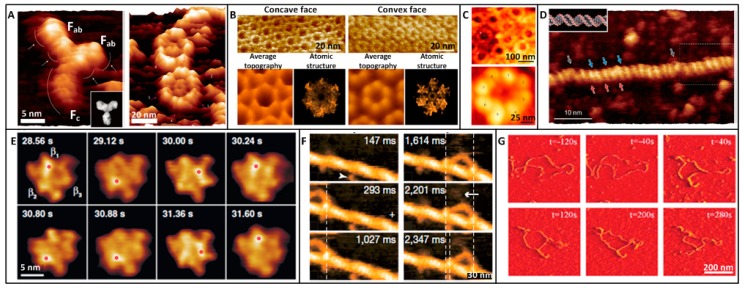
Imaging the static and dynamic structures of individual biological molecules under physiological conditions by AFM. (**A**–**D**) Static imaging. (**A**) IgG antibody molecule. High-resolution AFM image of anti-HSA mouse monoclonal antibody (IgG) adsorbed on a mica and self-assembled antibody hexamers composed of six IgG molecules. Images were recorded in 50 mM ZnCl_2_ and 50 mM MgCl_2_ solution respectively. Reprinted with permission from [58]. Copyright 2014 Macmillan Publishers Limited. (**B**) Bacterial microcompartment protein. The concave face has a depression diameter of 52.8 angstrom whereas the convex face has a diameter of 47.1 angstrom measured by AFM cross-section analysis. Reprinted with permission from [59]. Copyright 2015 American Chemical Society. (**C**) Nuclear pore complexes (NPCs). Numerous NPCs in the cytoplasm-facing outer nuclear membrane. Average projected structure of a vacant NPC showing eight cytoplasmic filaments that surround a central pore. Reprinted with permission from [60]. Copyright 2016 Macmillan Publishers Limited. (**D**) DNA. The red and blue arrows indicate the positions of major and minor grooves of B-DNA, respectively. Gray arrows indicate the local melting regions of the plasmid DNA. Reprinted with permission from [61]. Copyright 2013 American Chemical Society. (E–G) Dynamic imaging. (**E**) Rotary motor molecule. Successive AFM images showing the conformational change of β subunits in ATP. The highest pixel in each image is indicated by the red circle. Frame rate, 12.5 frame/s. Reprinted with permission from [39]. Copyright 2011 AAAS. (**F**) Walking myosin molecule. Successive AFM images showing the processive movement M5-HMM in ATP. Arrows indicate coiled-coil tail of M5-HMM tilted towards the minus end of actin. Reprinted with permission from [12]. Copyright 2010 Macmillan Publishers Limited. (**G**) DNA after drug stimulation. AFM images of the conformational changes of single DNA induced by the injection of Dau. Reprinted with permission from [63]. Copyright 2013 American Chemical Society.

Imaging the topography of single native biological molecules under physiological conditions is one of the most important applications of AFM in single-molecule assay. Antibodies are protein molecules that play a critical role in humoral immunity. The antibody structure has been revealed by electron microscopy and X-ray crystallography under non-physiological conditions, which cannot faithfully reflect the real situation. In 2014, Ido et al. [58] acquired the high-resolution topography of single IgG molecules in aqueous condition by AFM, as shown in Figure 2A. IgG molecules were immobilized onto a freshly cleaved mica in 50 mM ZnCl_2_ solution. In order to obtain high quality AFM images of single molecules, the crucial prerequisite is to immobilize the molecules firmly to a supporting surface so that the position of the probe with respect to the sample can be defined with high precision during imaging. Mica is an idea supporting surface, since the surface of freshly cleaved mica provides atomically flat surfaces over large areas [19]. Mica is negatively charged in aqueous solution, and thus mica surface facilitates electrostatic adsorption of positively charged molecules. For negatively charged molecules, they can adhere to the mica surface in cation solution. Hence the buffer solution containing cations is a key to successful imaging. In [58], IgG molecules are anchored to mica surface firmly in Zn^2+^ solution. The obtained AFM image clearly showed the Y-shaped structure of individual IgG molecule. Besides, monoclonal IgG molecules could self-assemble into well-ordered hexamers in aqueous solution. The Fc regions formed a doughnut-like inner assembly area at the center of the hexamer, and the outer petal-like structures were composed of six pairs of Fab regions.

Bacterial microcompartments (BMCs) are proteinaceous organelles that are widespread among bacterial phyla. In 2016, Sutter et al. [59] revealed the detailed topography of native BMC shell hexamers in solution by AFM, as shown in Figure 2B. BMC proteins from *Haliangiumochraceum* were adsorbed to the mica in buffer (50 mM Tris-HCl, pH 7.8, 100 mM NaCl, 10 mM MgCl_2_). Two distinct surface morphologies of the hexamer patches were discerned in AFM topography images, corresponding to the concave and convex faces of the hexamers observed in crystal structure. Further, the dynamics of BMC sheet formation were observed by time-lapse AFM imaging. Besides the molecules on the cell surface, AFM can also visualize the intracellular structures by isolating them from the cell. Nuclear pore complexes (NPCs) are biological nanomachines that mediate the bidirectional traffic of macromolecules between the cytoplasm and nucleus in eukaryotic cells. In 2016, Sakiyama et al. [60] clearly visualized the NPC structure in buffer solution by AFM, as shown in Figure 2C. Nuclei from *Xenopuslaevis* were adsorbed onto a poly-l-lysine-coated glass in low salt buffer (LSB) (1mM KCl, 0.5 mM MgCl_2_, 10 mM Hepes, pH 7.5). Poly-l-lysine is positively charged and cells are negatively charged. Hence substrate coated by poly-l-lysine can capture cells via electrostatic adsorption. AFM images were obtained in LSB solution. Pore-to-pore variability was evidently observed and about 40% of all NPCs showed large ‘plug-like’ features which were the cargoes caught in transit. By time-lapse imaging, the nanoscopic spatiotemporal dynamics of phenylalanine-glycine nucleoporins (FG Nups) proteins inside individual NPCs were revealed, providing novel insights into the dynamics of FGNups in NPC transport at the single-molecule level. In 2013, Ido et al. [61] clearly observed the helix structures of single native DNA in water by AFM, as shown in Figure 2D. DNA was adsorbed onto a freshly cleaved mica in 50 mM NiCl_2_ solution. Two distinct types of grooves with different widths appeared alternately, which corresponded to the major and minor grooves of B-form DNA.

Besides obtaining the static topography of single native biological molecules, the dynamic conformational changes of single molecules can be captured with the use of high-speed AFM. F_1_-ATPase is an adenosine triphosphate (ATP)-driven motor in which three torque-generating β subunits in the α_3_β_3_ stator ring sequentially undergo conformational changes upon ATP hydrolysis to rotate the central shaft γ unidirectionally. In 2011, Uchihashi et al. [39] recorded the dynamic structural changes of single F_1_-ATPase molecule, as shown in Figure 2E. The α_3_β_3_ subcomplex was covalently immobilized on mica. Mica was treated by 3-aminopropyltriethoxysilane and glutaraldehyde. Then a droplet containing α_3_β_3_ subcomplex was deposited on the surface. AFM images were recorded in buffer solution (10 mM Tris-HCl, pH 8.0, 2 mM MgCl_2_). In the absence of nucleotide, the α_3_β_3_ showed a pseudo-sixfold symmetric ring in which three alternately arranged subunits were elevated relative to the other three. When a non-hydrolyzable ATP was added, the ring became triangular and the central hole was obscured. By obtaining successive AFM images, the dynamic conformational changes of single α_3_β_3_ after the addition of ATP were clearly obtained. In 2010, researchers from the same group [12] investigated the dynamic walking process of single myosin molecule. The mica surface was firstly covered with biotin-containing lipid bilayers and then streptavidins were deposited on the substrate. Biotinylated actin filaments were immobilized on the bilayer surface through streptavidin molecules. Successive AFM images clearly showed that single myosin molecule moved processively along the actin filaments with discrete 36-nm steps.

DNAs have been widely imaged by AFM [62], and investigating the dynamic DNA-drug interactions provides a new idea to understand drug actions at single-molecule level. In 2013, Alonso-Sarduy et al. [63] investigated the dynamic conformational changes of single plasmid DNAs after the stimulation of chemotherapy drug Dauin aqueous condition, as shown in Figure 2G. For imaging DNA in liquids, the [Mg^2+^]/[Na^+^] ratio was crucial for the establishment of optimal DNA-imaging conditions. DNA molecules were immobilized to mica surface via 2 mM MgCl_2_ and 10 mM NaCl. Successive AFM images showed that the degree of negative supercoiling decreased and then reversed to a positive supercoiling and local plectonemic strands formed. Notably, for directly attaching DNAs onto mica in solution containing cation, DNAs form random shapes on mica. Recently, Endo et al. [64] have developed an observation scaffold based on the DNA origami structure. The scaffold can accommodate two DNA in its cavity to control the physical properties of the DNA (such as tension). With the method, diverse DNA-related molecular interactions (e.g., DNA methyltransferase, DNA repairenzymes) have been observed at single-molecule level, providing a novel way to precisely investigate the molecular interactions. These experimental studies demonstrated the unique capability of AFM in characterizing the topography of single native biological molecules (e.g., antibody, membrane protein, nuclear protein, DNA) at work under near-physiological conditions and monitoring their conformational changes, providing visual evidence for the behaviors of single molecules. Besides, the studies offer outstanding templates for investigating the diverse other types of biological samples and phenomena [37], which will be particularly useful for us to understand the biological activities at the single-molecule level.

## 4. Molecular Mechanics

**Figure 3:**
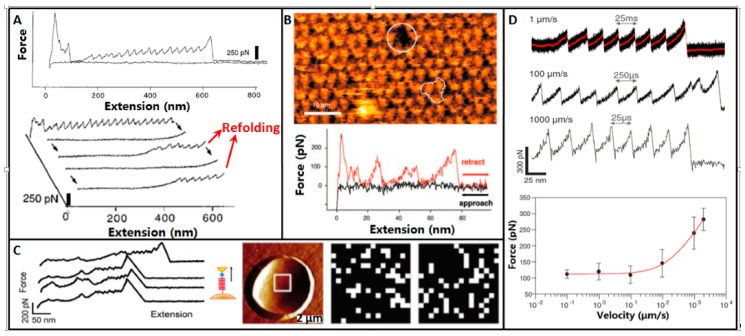
Unfolding single proteins by AFM. (**A**) Titin molecule. Force extension curves obtained by stretching titin proteins show periodic features that are consistent with their modular construction. Repeated stretch-relaxation cycles of single titin fragments demonstrate refolding. Reprinted with permission from [66]. Copyright 1997 AAAS. (**B**) Bacteriorhodopsin membrane protein. The tip and protein surface was separated at a velocity of 40 nm/s while the force spectrum was recorded. After the force extension curve was recorded, a topography of the same surface was taken to show structural changes. Note that a single monomer is missing (white circle). Reprinted with permission from [25]. Copyright 2000 AAAS. (**C**) Membrane protein on live cells. Force extension curves recorded between an Ig-T tip and surface of yeast cells expressing Als5p. Cells are trapped in porous membrane. Unfolding forces on cell surfaces were mapped by recording arrays of force extension curves on 500 nm × 500 nm areas. Reprinted with permission from [69]. Copyright 2009 American Chemical Society. (**D**) High-speed unfolding of titin molecule. Typical force extension curves are recorded at different retraction velocities (1, 100, 1000 µm/s) and dynamic force spectrum of the intermediate unfolding state. Solid red line is the theoretical fitting. Reprinted with permission from [71]. Copyright 2013 AAAS.

Besides directly visualizing the topography structures of single proteins, AFM can also mechanically reveal the dynamic information in the unfolding process of single protein. Protein molecules acquire their functions by specifically folding their polypeptide chains into well-defined three dimensional structures, and thus the unfolding information becomes a “molecular fingerprint” for indicating the proteins [65]. In 1997, Rief et al. [66] firstly used AFM to mechanically unfold single titin molecules, as shown in Figure 3A. Titins were allowed to adsorb onto gold surface in phosphate buffered saline (PBS) solution. The force extension curves evidently exhibited a sawtooth-like pattern, with a periodicity that varied between 25 and 28 nm. Force extension curves of the same titin molecule were recorded to examine the refolding of titin. After each extension, the molecule was allowed to relax completely. A completely cycle took approximately 1 s. The subsequently recorded force extension curves also exhibited sawtooth-like peaks, but the peaks were fewer. The results showed that titin refolded but only a fraction of the domains refolded. During the process of unfolding a molecule, it was possible that the molecule was fully unfolded but did not detach from the cantilever tip. Tension on the protein might then be relaxed by returning the tip to the position before unfolding [67]. In this case, the domains of the protein might actually refold, allowing the protein to undergo forced unfolding again. The research demonstrate that AFM could investigate the unfolding and refolding process of single proteins.

Then researchers explored utilizing AFM to directly unfold single membrane proteins which were trapped in the cytomembrane. In 2000, Oesterhelt et al. [25] used AFM to unfold single native membrane protein molecules, as shown in Figure 3B. Purple membrane is a special patch of the cell membrane of *Halobacteriumsalinarum*. In the purple membrane, there is only one type of protein called bacteriorhodopsin (BR) [68]. Hence, BR in the purple membrane is an ideal sample for AFM studies. The native purple membrane patches isolated from *Halobacteriumsalinarum* were allowed to adsorb to mica surface. The well-pronounced single BRs in the purple membrane were visualized by AFM imaging. Then AFM tip was located to single BR and unfold it. The force extension curve showed specific sawtooth-like peaks, which corresponded to the unfolding of BR. For verification, AFM imaging was performed at the same area again and the image showed a distinct hole in the position of the unfolded BR.

AFM can not only mechanically unfold single proteins adsorbed on mica or reconstituted in the membrane, but also can directly unfold single membrane proteins on the surface of live cells. In 2009, Alsteens et al. [69] applied AFM to unfold single Als5p proteins directly on live cells, as shown in Figure 3C. Single *Saccharomyces cerevisiae* cells (a species of yeast) expressing Als5p were mechanically trapped in porous polycarbonate membranes (the pore size in the polycarbonate membranes was similar to the cell size). Ig-T molecules were attached to the surface of AFM tip which was previously coated by a layer of gold. Force extension curves were recorded when indenting the cell surface with Ig-T-linked AFM tip. The force extension curves displayed distinctly sawtooth-like patterns, which corresponded to the unfolding dynamics of Als5p proteins. By recording arrays of force extension curves on the cell surface, the gray maps reflecting the unfolding force of Als5p were constructed.

The unfolding of single protein by conventional AFM had a slow pulling velocity (e.g., 40 nm/s [25,70]), which was much smaller than the molecular dynamics simulations. In 2013, Rico et al. [71] applied high-speed AFM to unfold single titin molecules at high pulling velocities, as shown in Figure 3D. Force extension curves were acquired at pulling velocities ranging over six orders of magnitude, from 0.0097 to 3870 μm/s. At slow velocities, the force extension curves were in agreement with the results by conventional AFM. But when the pulling velocities were higher than 100 μm/s, the unfolding forces followed a significantly steeper slope, which was consistent with the results obtained by molecular simulations. The research enabled unfolding single proteins in microsecond time resolution which was comparable to molecular simulations and thus allowed direct comparison of experimental and simulated unfolding results.

These experimental results (Figure 3) demonstrate that AFM can mechanically unfold different types of protein molecules (including isolated proteins adsorbed on mica [66], native proteins reconstituted on membrane patch [25,49], native proteins on live cell [69]) in aqueous conditions at the single-molecule level and the unfolding timescale can be comparable to that in molecular dynamic simulations [71], providing an invaluable system for experimentally exploring the specific mechanical behaviors of single proteins. With the established method, other types of proteins can be investigated, which will be particularly useful in understanding fundamental mechanisms guiding the behaviors of single molecules, e.g., proteins folding pathway, how proteins tune their functions by different folding pathways, false folding and disease, and how to tune protein folding.

## 5. Molecular Recognition

**Figure 4:**
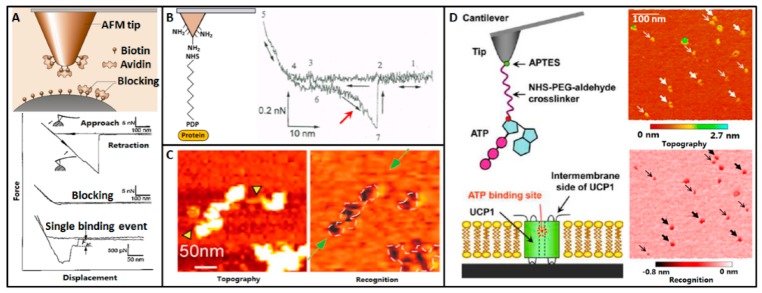
Detecting and recognizing individual receptor-ligand events by AFM. (**A**) Biotin-avidin. Force curves are recorded on biotinylated bead by avidin-functionalized tip. The specificity is demonstrated by blocking with excess free avidins. Single unbinding event is measured by decreasing the density of biotins on bead. Reprinted with permission from [72]. Copyright 1994 AAAS. (**B**) Antibody-antigen. The antibody is linked to AFM tip via PEG molecule. Force curve recorded on antigen-coated substrate exhibits a significant molecular unbinding peak. Reprinted with permission from [73]. Copyright 1996 National Academy of Sciences. (**C**) TREC imaging of nucleosomes on mica. Topography image and recognition image is recorded simultaneously by the antibody-functionalized AFM tip. White pixels in topography image are the nucleosomes and black pixels in recognition image are the recognition signals. Reprinted with permission from [77]. Copyright 2004 National Academy of Sciences. (**D**) TREC imaging of proteins reconstituted in lipid bilayer. TREC imaging on UCP1-reconstituted lipid bilayer is performed using ATP-functionalized tip. Reprinted with permission from [78]. Copyright 2013 American Chemical Society.

Using AFM to specifically probe the target molecules on the surface is an important and successful application of AFM in single-molecule assay. In 1994, Florin et al. [72] firstly used AFM to measure the unbinding force of individual molecular pair (biotin-avidin), as shown in Figure 4A. AFM tip was firstly coated by a layer of biotinylated bovine serum albumin (BSA) and then incubated with avidin molecules. The AFM tip was moved to biotinylated agarose beads to obtain force curves. The obtained force curves clearly showed the adhesion peaks caused by the rupture of biotin-avidin pair. After adding the avidins to block the biotins on the beads, there were no adhesion peaks in the obtained force curves, demonstrating the specific biotin-avidin binding during force spectroscopy. But the adhesion peaks in the obtained force curves contained as many as 100 biotin-avidin pairs. By blocking most of the biotin on the beads, the number of interacting molecules were significantly reduced and the single binding events were measured.

In 1996, Hinterdorfer et al. [73] measured single antibody-antigen recognition events by linking antibodies to the surface of AFM tip via a polyethylene glycol (PEG) spacer molecule, as shown in Figure 4B. The length of the PEG molecule was about 8 nm. Antibodies were linked to the surface of AFM tip. The antigen proteins were adsorbed onto mica by the same PEG spacer molecule. The density of antibodies on tips was adjusted to best meet the expectation that only one antibody might interact with the mica surface. From the force curve, there was a significant non-linear unbinding peak (denoted by the red arrow in Figure 4B), which corresponded to the stretching of the PEG linker and the rupture of the antigen-antibody bond. The unbinding peak vanished after adding free antibodies to block the antigens on the mica surface, demonstrating the specificity of antibody-antigen interaction events.

Linking proteins onto the AFM tip via PEG spacer molecules have several advantages. First, the PEG is covalently bound to both the tip and the antibodies, and the covalent bond is much stronger than the receptor-ligand bond [6]. During the retraction the receptor-ligand bond ruptures first, which ensures that the unbinding peak is associated only with the receptor-ligand binding. Second, the PEG linker allows the ligand to freely reorient to interact with receptors and avoids compressing the receptors [53]. Third, the PEG linker allows a clear distinction between specific and unspecific molecular binding because of the soft and non-linear elasticity of the PEG linker [74]. There are many types of PEG spacer molecules, such as NHS-PEG-pyridyldithiopropionyl (PDP) [73], NHS-PEG-maleimide (MAL) [75], and NHS-PEG-aldehyde [76]. For tip functionalization, the AFM tip was firstly coated by a layer of NH_2_. The NHS end of the PEG linker can covalently bind to the NH_2_ on the tip surface. The PDP (MAL) end of the PEG linker can covalently bind to the thiol groups of the protein. Alternatively, the proteins can be linked to the aldehyde end of the PEG via the lysines in the proteins [53]. Antibodies do not have thiol groups, and thus antibodies are often treated by N-succinimidyl3-(acetylthio)propionate (SATP) to form thiol groups [76].

By scanning the substrate coated by biological molecules with functionalized tip at TREC mode, the specific recognition information of the biological molecules can be rapidly acquired. In 2014, Stroh et al. [77] investigated the nucleosomes on mica by TREC imaging, as shown in Figure 4C. Mica was treated by glutaraldehyde and 3-aminopropyltriethoxysilane. The nucleosomal arrays adsorbed onto the surface of the treated mica. Polyclonal anti-histone H3 antibodies were thiolated and linked to the surface of AFM tip via PEG linker. Images were acquired in PBS. The topography image and recognition image was simultaneously recorded. The dark pixels in the recognition image corresponded to the specific recognition information. The recognition patches and the pattern in which they occurred clearly coincided with the positions of the nucleosomes in the topography image, demonstrating the validity of TREC imaging in recognizing specific molecules. TREC imaging can not only recognize the molecules on mica, but also can recognize the molecules in lipid bilayers. In 2013, Zhu et al. [78] investigated the proteins in lipid bilayer by TREC imaging, as shown in Figure 4D. The uncoupling protein 1 (UCP1) was reconstituted in bilayers on mica. ATPs were linked to AFM tip via NHS-PEG-aldehyde spacer molecule. Images were acquired in buffer solution. The recognition information (dark pixels) in the recognition image was fully consistent with the positions of proteins in the topography image. Compared with directly adsorbing proteins onto mica surface, reconstituting proteins in lipid bilayer can better mimic the proteins in cytomembrane and thus is useful in investigating the behaviors of single proteins.

## 6. Molecular Activities on Cell Surface

**Figure 5:**
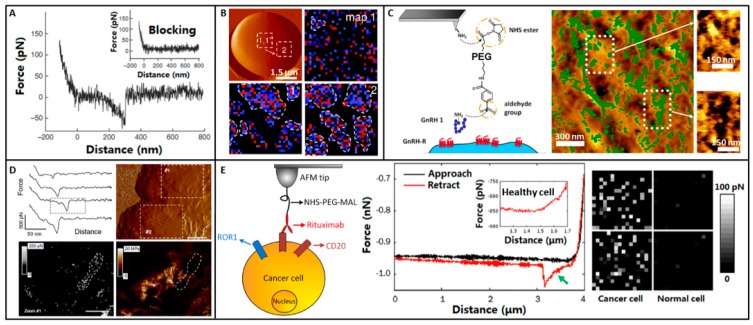
Detecting individual receptors on cell surface. (**A**) Recognition of SGLT1 on the surface of intact cells by AFM tip coated with specific antibodies. Typical force curve shows specific interaction between the antibody and SGLT1 upon tip-surface retraction. The specific interaction is blocked by adding free antibodies to the solution (inset). Reprinted with permission from [89]. Copyright 2011 Macmillan Publishers Limited. (**B**) Formation and propagation of Als5p nanodomains. Blue andred pixels correspond to forces smaller and larger than 150 pN, respectively, and thus to Als5p recognition and unfolding. Reprinted with permission from [90]. Copyright 2010 National Academy of Sciences. (**C**) Location of the GnRH-Rs on the T24 cell surface by TREC imaging. Overlays of recognition maps of GnRH-Rs onto the corresponding topography images. Reprinted with permission from [91]. Copyright 2014 American Chemical Society. (**D**) Bacteriophage extrusion localizes into soft nanodomains detected by PFT imaging. The force curves recorded during PFT imaging indicate that the phage-cell wall complex behaves as a Hookean spring. Topography image, adhesion image and elasticity image is recorded simultaneously. Reprinted with permission from [92]. Copyright 2014 Macmillan Publishers Limited. (**E**) Molecular recognition on primary tumor cell from clinical lymphoma patients. Tumor cells from bone marrow sample are recognized by ROR1 fluorescence labeling. CD20-rituximab interactions are detected on tumor cell. There is a specific unbinding peak (green arrow) in the force curve recorded on tumor cell but not in the force curve recorded on healthy cell. The distributions of CD20s on tumor cells are mapped by obtaining arrays of force curves on 500 × 500 nm^2^ areas. Reprinted with permission from [93]. Copyright 2013 Elsevier Inc.

Compared with probing molecules immobilized on substrate, directly probing the single molecules on the surface of cells can better reflect the real situations [79,80] and is useful in helping us to understand the molecular behaviors on cell surface. In the past decades, researchers have used AFM-based SMFS to widely probe the specific molecules on live cells at the single-molecule level, such as the heat shock protein [81], transporter [82], growth factor receptor [83], glycoprotein on glioblastoma cell [55], antigen on lymphocyte [84], fibrinogen receptor on erythrocyte [85], adhesion molecules on microbial cell [86], Fc receptors on macrophage [87], and cell-cell adhesion molecules [88]. For probing the individual specific receptor molecules on the living cells, attaching the ligand (or antibody) molecules that can specifically bind to the receptor molecules is crucial. The main steps involved in SMFS studies on living cells include positioning AFM tip above cell monolayers, cell imaging with uncoated tip, performing force curves on cell surface (force curves are obtained at different positions on cell surface and many cells are tested), blocking (blocking the AFM tip or the receptor molecules on cell surface), performing force curves again, and data analysis [89]. Figure 5A is a typical force curve obtained on living G6D3 cell with antibody-functionalized tip. The antibody on the AFM tip could specifically bind to the transporter molecule SGLT1 on cell surface. There was a distinct unbinding peak in the force curve. After blocking, the specific unbinding peak vanished, proving the specific transporter-antibody interactions.

In 2010, Alsteens et al. [90] investigated the single adhesion molecules on the surface of live microbial cells, showing that pulling on single adhesins with AFM tips functionalized with specific antibodies triggered the formation of adhesion domains of 100–500 nm and that the force-induced nanodomains propagated over the entire cell surface, as shown in Figure 5B. *Saccharomyces cerevisiae* cells were mechanically trapped in porous polycarbonate membranes whose pore size was similar to the cell size. AFM tips were functionalized with anti-V5 antibodies via NHS-PEG-acetal linker. The first adhesion force map (32 × 32 force curves on 1 × 1 μm^2^ area) obtained on the cell surface revealed that the proteins were evenly distributed. The second adhesion force map obtained on the same area exhibited that the proteins clustered. The adhesion force maps obtained on remote area localized several hundred nanometer away also showed that proteins clustered. Further experiments showed that after the formation of domains, the domains propagated at a speed of about 20 nm/min on the cell surface.

In 2014, Zhang et al. [91] applied TREC imaging to investigate the human gonadotropin-releasing hormone receptor (GnRH-R) on chemically fixed T24 cells, as shown in Figure 5C. Ligands were covalently bound onto AFM tips via NHS-PEG-acetal linker. By TREC imaging, topography image and recognition image were simultaneously acquired. The imaging showed that the recognition spots were irregularly distributed on the cell surface, and most of them were located on the high features of the T24 cell surface. Statistical analysis of recognition images revealed that GnRH-Rs mostly formed nanodomains with areas from about 100 to 28,000 nm^2^. By using PFT imaging with functionalized tips, the structural, adhesion, and elasticity information can be correlated. In 2013, Alsteens et al. [92] investigated the single bacteriophages extruding from living bacteria, as shown in Figure 5D. Bacteriophages were genetically engineered to display His-tag groups and AFM tip was functionalized with Ni^2+^-NTA groups to detect single bacteriophages. The real-time recorded force curves showed the specific molecular interaction events. AFM topography image, adhesion image, and elasticity image were simultaneously acquired, showing that bacteriophages were preferentially detected in soft nanodomain regions of the bacteria and these soft nanodomains were surrounded by stiffer cell wall material.

Currently, AFM-based SMFS studies are commonly performed on cell lines cultured in vitro, which are quite different from the cells in the human body. Directly probing the molecular activities on primary cancer cells from patients can better reflect the real situations. We have used AFM to probe the CD20 molecules on the surface of primary lymphoma cells from clinical patients [93], as shown in Figure 5E. For B-cell lymphoma patients with bone marrow invasion, the obtained bone marrow biopsy contains both lymphoma cells and healthy cells. Hence, the prerequisite is to recognize the lymphoma cells in the bone marrow sample. Recent studies have shown that the receptor tyrosine kinase-like orphan receptor 1 (ROR1) is selectively expressed on the surface of B-cell chronic lymphocytic leukemia and on some B-cell lymphomas (including mantle cell lymphoma, marginal zone lymphoma, follicular lymphoma), whereas normal B cells and other normal cells do not express ROR1 [94,95]. Hence, ROR1 is a suitable marker for distinguishing tumor B cells from healthy cells. By ROR1 fluorescence labeling, tumor B cells were recognized and then the AFM tip carrying rituximab (a monoclonal anti-CD20 antibody) was moved to tumor B cells to probe the CD20s on cell surface. There were specific unbinding peaks in the retract curve (denoted by the green arrow in Figure 5E) obtained on tumor B cells, while there were no specific unbinding peaks in the retract curve obtained on normal cells. By obtaining arrays of (16 × 16) force curves on local areas (500 × 500 nm^2^), the distributions of CD20s on cell surface were mapped. The gray pixels in the maps visually reflected the distribution of CD20s on lymphoma cell, whereas the maps on normal cells did not exhibit recognition information. The distributions of CD20s on tumor B cells for three lymphoma patients were measured and were combined with the clinical therapeutic outcomes, preliminarily showing that to some extent the distributions of CD20s on tumor cell surface were related to the clinical rituximab therapy outcomes [96]. The research established the procedure of detecting the target proteins on tumor B cells from clinical B-cell lymphoma patients with bone marrow invasion and demonstrated the capability of AFM-based SMFS in probing human primary tumor cells, providing novel insights into molecular biophysical properties and behaviors in near-in vivo conditions and offering a new way to explore potentially meaningful biomarkers for biomedical applications.

## 7. Challenges and Outlook

AFM has achieved great success in detecting the behaviors of single molecules in diverse fields, including molecular imaging, molecular mechanics, molecular recognition, and molecular activities on cell surface, considerably contributing to cell and molecular biology. However, there are still many challenges needing to be addressed.

The first challenge is how to detect individual molecules on living cells. Current AFM-based single-molecule studies are commonly performed on purified molecules [12,39,58,78,97] or on chemically fixed cells [91,93]. Though high-speed AFM and multiparametric AFM significantly improves the performance of AFM, these two techniques are mainly suited for samples with flat (such as molecules adsorbed on mica [12,39]) and rigid (such as microbial cell [92] which has stiff cell wall and some specific types of eukaryotic cells [26,42]) surface. Currently, the individual molecules on the surface of living mammalian cells cannot be observed [14]. This is mainly due to the soft and dynamic nature of the cell membrane, which causes the deformation of the membrane when AFM tip contacts cell membrane and then results in the decrease of imaging resolution. Recently, researchers have investigated AFM imaging with adaptive contact mode, which could acquire AFM image with smaller probe-cell interaction force [98], providing a new idea to realize the high-resolution imaging on living cells. Conventionally the contact mode imaging uses a fixed set-point of cantilever deflection. In this situation, the force applied may be exceedingly large for areas where the sample is relatively flat, whereas not large enough to maintain the image quality at areas where the sample topography variation is large. By integrating a gradient-based optimization scheme which could adaptively adjust the cantilever deflection set-point line-by-line to the feedback control system, the normal force was maintained around the minimal level throughout the entire imaging process and thus scanning at near-minimum-force was realized.

In order to probe the individual target molecules on cell surface, AFM tips are functionalized with antibodies that can specifically bind to the target molecules on cell surface. However, it is often difficult to differentiate the nonspecific binding events from specific interactions owing to the multiple types of molecules on cell surface [99]. Besides, when performing SMFS experiments on living cells, studies have shown that the target molecules can be unfolded by the pulling tip [69]. Hence, several concerns may appear, such as whether pulling the target molecule can cause the conformational changes of the target molecule and the possible influence on cellular activities. Information about these concerns is still scarce. The distributions of target molecules on the cell surface can be mapped by obtaining arrays of force curves [93] or TREC mode [91]. However, because the PEG linkers can orient freely, there may be some target molecules that are ignored by the antibodies on AFM tips and also some target molecules that are repeatedly detected. Besides, the curvature radius of AFM tip (~10 nm) is much larger than the size of individual molecule (<2 nm [25]). Hence, it is challenging to locate the exact positions of individual molecules on cell surface by AFM. Further studies are needed to address these issues. For example, we can combine AFM force spectroscopy with single-molecule fluorescence technology [11] to examine whether pulling the fluorescein-labeled target molecules on the living cells can result in the conformation changes of target molecules (the conformational changes can be detected via fluorescence). In addition, the detailed distribution of target molecules on cell surface can be visualized by single-molecule fluorescence microscopy [100], which can then be compared with the results detected by AFM.

The second challenge is the correlation between AFM detected information and molecular/cellular behaviors. AFM can acquire multiple types of information about the physical properties of individual molecules, such as morphology, unfolding/refolding mechanics, binding affinity, and distributions on cell surface, whereas knowledge about molecular activities is commonly deduced from ensemble measurements by biochemical experiments. Hence, the challenge is to determine which parameters acquired by AFM contain meaningful information about biological functions. At the single-cell level, information obtained by AFM such as cell mechanical properties has been shown to be potential biomarkers for indicating cell states [101]. Whether the acquired information by AFM at the single-molecule level can serve as a potential biomarker for indicating the states of molecules or diseases is still to be investigated. For further studies, AFM should be combined with complementary tools such as fluorescence microscopy [37] to make it possible to correlate the AFM-obtained information and functions of molecules.

The third challenge is the standardization of AFM single-molecule experiments. The outcome of an AFM experiment is related to several factors, e.g., sample preparation, tip preparation, data collection and interpretation [97], and the experience of the experimenter. Especially in force spectroscopy, current procedures for linking biological molecules to AFM cantilevers are often complex [53]. Besides, the functionalized tip may lose activities due to tip damage or contamination, especially when performing experiments on living cells in culture medium [97]. For example, the conformation of ligands on AFM tip may be changed due to the frequently mechanical touching between AFM tip and sample surface, which may then result in that the ligands do not bind to the receptors on cell surface. Controlling the contact force between AFM tip and sample to a low level (such as less than 100 pN [18]) may reduce the risk of tip contamination. For molecular imaging, often mica is treated to adsorb target molecules, such as proteins and DNAs. Especially when imaging molecules in water, molecules need to be immobilized on the mica surface and this immobilization should not be too strong to inhibit the changes of molecular conformations. Especially in some cases, specific substrates (such as mica-supported lipid bilayers and streptavidin 2D crystals) [102] are used for observing the dynamics of single molecules by high-speed AFM. Consequently, defining standardized protocols for AFM single-molecule experiments will make AFM more appealing to researchers.

In summary, the applications of AFM single-molecule assay have made significant contributions in understanding the behaviors of individual molecules. Addressing the challenges facing AFM-based single-molecule techniques will require the efforts of researchers from different disciplines. As AFM is utilized to investigate more biological systems, we have much to look forward to.

## Appendix A

**Table A1:** AFM probes for single-molecule sensing.

Typical Applications	Tip Material	Tip Radius	Cantilever Spring Constant	References
Antibody imaging on mica	Silicon	<10 nm	42 N/m	[58]
Membrane protein imaging on mica	Silicon nitride	≤20 nm	0.09 N/m	[59]
High-speed imaging of molecules	Silicon nitride	~4 nm	0.1–0.2 N/m	[12]
High-speed imaging of living cell	Silicon nitride	<10 nm	0.1 N/m	[44]
Imaging DNA dynamics in water	Silicon nitride	<10 nm	0.32 N/m	[63]
Unfolding single proteins on cells	Silicon nitride	<10 nm	0.01–0.025 N/m	[69]
High-speed unfolding proteins	Silicon nitride	<10 nm	~0.1 N/m	[71]
SMFS on cell surface	Silicon nitride	~10 nm	0.008–0.021 N/m	[90]
TREC imaging on cell surface	Silicon	8 nm	0.14 N/m	[91]
PFT multiparametric imaging	Silicon nitride	~20 nm	0.45–0.77 N/m	[92]
